# Western diet promotes the progression of metabolic dysfunction‐associated steatotic liver disease in association with ferroptosis in male mice

**DOI:** 10.14814/phy2.70139

**Published:** 2024-11-28

**Authors:** Nicole Maddie, Nefia Chacko, David Matatov, Maria Alicia Carrillo‐Sepulveda

**Affiliations:** ^1^ Department of Biomedical Sciences, College of Osteopathic Medicine New York Institute of Technology Old Westbury New York USA

**Keywords:** ferroptosis, GPX4, MASH, MASLD, obesity, Western diet

## Abstract

Non‐alcoholic fatty liver disease (NAFLD), now referred to as metabolic dysfunction‐associated steatotic liver disease (MASLD), is a silent killer that often progresses to metabolic dysfunction‐associated steatohepatitis (MASH). To date, there are no pharmacological treatments for MASLD. While obesity is a major cause of the development and progression of MASLD, the underlying mechanisms remain unclear. We hypothesize that ferroptosis, a recently discovered nonapoptotic iron‐dependent form of cell death, is activated during the progression of MASLD and may be a potential target for treating MASLD. Using a murine model of Western diet‐induced obesity, C57BL/6J male mice were exposed to a long‐term (36 weeks) Western diet. Controls were maintained with a standard chow diet. Western diet‐induced obesity was confirmed by increased body mass index (BMI). Histopathological analysis demonstrated the progression of MASLD to MASH in the obese group, which was accompanied by significant hepatic iron deposition, oxidative damage, and lipid peroxidation. Hepatic ferroptosis was further confirmed by decreased protein expression of glutathione peroxidase 4 (GPX4) and increased acyl‐CoA synthetase long‐chain family member 4 (ACSL4), markers of ferroptosis. These findings suggest that ferroptosis is a potential mechanism involved in the pathogenesis of MASLD in male mice.

## Graphical abstract

The graphical figure illustrates that long‐term Western diet‐induced obesity which in turn leads to (1) iron accumulation and (2) increased expression of ACSL4 and decreased expression of GPX4, markers of ferroptosis, in the livers. These effects were followed by hepatic (3) oxidative damage and (4) lipid peroxidation, events well‐known to lead to (5) ferroptosis and ultimately to (6) MASH.

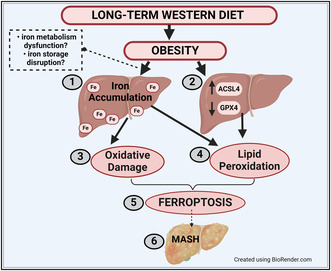

## 1 INTRODUCTION

Non‐alcoholic fatty liver disease (NAFLD) is considered a silent killer as it often remains asymptomatic before diagnosis (Siddiqui et al., 2013). It is estimated that one in four people are affected by NAFLD worldwide (Azzu et al., 2020; Cotter & Rinella, 2020). It has been considered a disease of modern society that encompasses Western lifestyles and eating habits.

Recently, a consensus of experts has redefined the diagnostic criteria of this disease to emphasize the metabolic dysfunction responsible for disease progression (Eslam et al., 2020). NAFLD, now referred to as metabolic dysfunction‐associated steatotic liver disease (MASLD), is defined as >5% steatosis and one of three metabolic derangements including overweight/obese, type 2 diabetes mellitus, or metabolic risk factors (Eslam et al., 2020; Gofton et al., 2023; Rinella et al., 2024). MASLD is characterized by excessive lipid accumulation within the liver, encompassing a histological spectrum of progressive liver conditions ranging from steatosis to steatohepatitis, severe fibrosis, and cirrhosis. (Azzu et al., 2020; Byrne & Targher, 2015).

A major contributor to the development and progression of MASLD is obesity, and it is also commonly associated with metabolic syndrome and insulin resistance (Azzu et al., 2020; Cotter & Rinella, 2020; Li et al., 2023; Younossi, 2019). There are currently no treatments to address obesity‐induced MASLD, and the prevalence continues to rise at an alarming rate (Qu et al., 2021; Younossi, 2019).

An emerging theory of MASLD development involves the process of ferroptosis. Ferroptosis is an iron‐dependent form of cell death that is inherently different from other forms of cell death such as apoptosis and necrosis (Feng et al., 2022). Ferroptosis induces cell death by depleting glutathione, decreasing glutathione peroxidase 4 (GPX4) activity, and generating large amounts of reactive oxygen species (ROS) (Zhang, Zhang, & Hu, 2021). Recently, it has been shown that patients with MASLD have iron accumulation in the liver, potentially linking ferroptosis to MASLD disease progression (Zhang, Zhang, & Hu, 2021). We hypothesize that ferroptosis is involved in the progression of MASLD in Western diet‐fed middle‐aged male mice.

## 2 METHODS

### 2.1 Animals and diet intervention

The incidence and prevalence of MASLD are significantly higher among men than women (Byrne & Targher, 2015). For this reason, experiments for this present study were performed on male C57BL/6J mice (Jackson Laboratories, Bar Harbor, ME, USA). Mice were kept in a temperature‐controlled facility of 21 ± 2°C and humidity of 50 ± 10% under a 12‐h light–dark cycle. They were given access to tap water and their respective diets ad libitum. At 12 weeks old, the mice were randomly assigned into two experimental groups: The control group (*n* = 7) was fed a standard chow diet (Lab Diet, Richmond, IN, USA, Cat.#. 5001) consisting of 14 kcal% fat, 57 kcal% carbohydrates (3.2% sucrose), and 29 kcal% protein with 240 ppm of iron and the obese group (*n* = 11) was fed a high‐fat, high‐carbohydrate Western diet (WD; OpenSource Diets, New Brunswick, NJ, USA, Cat.#. D12079B) consisting of 40 kcal% fat, 43 kcal% carbohydrates (34% sucrose), and 17 kcal% protein with 37 ppm of iron for 36 weeks. Based on our previously established model of Western diet‐induced obesity in female rats (Kramer et al., 2018), we utilized the same model to induce obesity in mice. Obesity was determined by increased body mass index (BMI), calculated as the ratio between body weight (g) and length (cm^2^), and the Lee Index, calculated as the cubic root of body weight divided by the nose‐to‐anus length (cm) (Cheng et al., 2021). The Lee Index was proposed for rodents with the same purpose as the human BMI. Body length was measured from nose to anus or nose to tail‐base length (Timotius et al., 2019). At the terminal experiments, mice were anesthetized with 5% isoflurane on 100% O_2_ flow. Termination was completed by exsanguination. Liver samples were quickly removed and stored immediately at −80°C for molecular analysis or in 10% formalin for histological analysis. All preclinical experimental protocols followed the ARRIVE guidelines and were conducted following the Institutional Animal Care and Use Committee at the New York Institute of Technology College of Osteopathic Medicine and for the National Institute of Health Guidelines for the Care and Use of Laboratory Animals (National Research Council Committee for the Update of the Guide for the and Use of Laboratory, 2011).

### 2.2 Analysis of metabolic parameters

Blood samples were obtained from the submandibular vein of unanesthetized mice, a method that minimizes mice distress. Serum alanine aminotransferase (ALT, Cat.#. A7526‐150) and aspartate aminotransferase (AST, Cat.#. A7561450) were determined by using analytical kits from Pointe Scientific (Canton, MI, USA) as per the manufacturer's instructions. Insulin resistance (IR) was assessed using the homeostasis model assessment (HOMA‐IR). It is calculated as [fasting insulin (μU/mL) × fasting glucose (mM)]/22.5 (Kiernan et al., 2022). Insulin levels were assessed by an ultrasensitive mouse insulin ELISA kit (Cat.#. 90,080, Crystal Chem, Elk Grove Village, IL) according to the manufacturer's instructions.

### 2.3 Intra‐peritoneal glucose tolerance test (IPGTT)

At 8 and 24 weeks into the dietary intervention, mice were fasted for 12 h before receiving an intraperitoneal injection of 20% glucose solution dosed at 2 g/kg body weight (Sigma, St. Louis, MO, USA). Blood samples were taken from tail veins immediately before (0 min) and at 5, 15, 30, 60, 90, and 120 min after glucose injection. Blood glucose levels were measured with an AimStrip Plus glucometer (Cat.#. 37,321, Germaine Laboratories, San Antonio, TX, USA).

### 2.4 Liver histology

Liver samples obtained at the terminal experiments were fixed in 10% formalin and then embedded in paraffin blocks. Hematoxylin–eosin (H&E) and Oil Red‐O (Cat.#. 01391, Millipore Sigma, Burlington, MA) were utilized to stain 10 μm‐thick liver sections. The NAFLD activity score (NAS), which reflects the sum of steatosis, lobular inflammation, and hepatocyte ballooning, was assessed by using the NASH Clinical Research Network histological scoring system algorithm on H&E‐stained livers (Pappachan et al., 2017). To assess NAFLD activity score, five different areas (× 200 magnification) were selected per mouse and analyzed by two researchers in a blinded manner. Steatosis was graded from a score of 0–3 defined as the following: <5% steatosis equates to 0, 5%–33% equates to 1, 34%–66% equate to 2, and >66% equates to 3. Lobular inflammation was evaluated based on a score of 0–4 by the following: no foci equate to 0, <2 foci per 200x field equate to 1, 2–4 foci per 200x field equate to 3, >4 foci per 200x field equate to 4. Hepatocyte ballooning was scored based on the following: no ballooned hepatocytes equate to 0, few ballooned hepatocytes equate to 1, and many/prominent ballooned hepatocytes equate to 2. The NAS score and subsequent NASH severity were assessed by the following: a score of 0–2 indicates no MASH, a score of 3–4 indicates borderline MASH, and a score of 5–8 indicates the presence of MASH (Pappachan et al., 2017).

To analyze lipid accumulation, some liver specimens were placed in an optimal cutting temperature (OCT) compound, rapidly frozen on dry ice, and stained with an Oil Solution. The lipid droplets were qualitatively assessed for differences between dietary protocols.

### 2.5 Iron staining

Iron content in the liver was determined utilizing a Prussian blue staining kit (Cat.#. ab150674, Abcam, Cambridge, MA, USA) following the instructions provided by the manufacturer's protocol (Chae et al., 2022; Wu et al., 2021). Liver cross sections in paraffin were stained for 30 min with Prussian blue and counterstained with nuclear solid red solution for 2 min. Staining Reading: Nuclei appear red, cytoplasm pink, and bright blue spots are iron. The staining intensity was graded from a scale of 0 to V: *Grade 0* (minimum grade), no staining; *Grade I*, small number of positive Kupffer cells; *Grade II*, moderate number of positive Kupffer cells; *Grade III*, most Kupffer cells are positive, with some positive hepatocytes, or no staining of Kupffer cells, but some positive hepatocytes; *Grade IV*, large number of positive Kupffer cells and hepatocytes; and *Grade V*, most hepatocytes and Kupffer cells are positive (Akatsu et al., 2020).

### 2.6 Western blot analysis

According to the instructions, protein content was determined in the supernatant of liver extract using a commercial BCA kit (Cat.#. 23,227, ThermoScientific, Rockford, IL, USA). Equivalent amounts of protein (50 μg per lane) from the livers of each experimental group were loaded and separated by 10% sodium dodecyl sulfate‐polyacrylamide gel electrophoresis (SDS‐PAGE) and then transferred to polyvinylidene difluoride (PVDF) membranes (Thermo Fisher Scientific Inc., Rockford, IL, USA), as previously described (Carrillo‐Sepulveda et al., 2015). Membranes were incubated overnight at 4°C with the following specific primary antibodies: anti‐acyl‐CoA synthetase long‐chain family member 4 (ACSL4; 1:1.000, Cat.#. sc‐365,230, Santa Cruz Biotechnology, Santa Cruz, CA, USA) and anti‐glutathione peroxidase 4 (GPX4; 1:1.000, Cat.#. ab125066, Abcam, Cambridge, MA, USA). Membranes were incubated with the corresponding secondary antibodies: anti‐rabbit (1:10.000, Cat.#. 7074S, Cell Signaling Technology, Danvers, MA, USA) and anti‐mouse (1:10.000, Cat.#. 7076S, Cell Signaling Technology, Danvers, MA, USA). The membranes were stripped and re‐probed with GAPDH (1:10.000, Cat.#. 2118, Cell Signaling Technology, Danvers, MA, USA) as a loading control. Protein expression was detected using Lumigen Enhanced Chemiluminescence (ECL) Ultra (Cat.#. TMA‐100; Lumigen Inc., Southfield, MI, USA). The data is presented as a percentage (%) of Control.

### 2.7 Hepatic oxidative damage analysis

Hepatic damage was determined by measuring oxidative damage and lipid peroxidation.

Oxidative damage was measured using 8‐OHdG immunostaining, a marker of RNA and DNA oxidative damage. Briefly, hepatic tissue sections were incubated at room temperature for 20 min in goat serum for blockage of nonspecific binding, followed by overnight incubation at 4°C with anti‐monoclonal antibodies against 8‐OHdG (1:100, Cat.#. sc‐66,036, Santa Cruz Biotechnology, Santa Cruz, CA, USA) and then incubated with secondary Alexa 488‐labeled goat antibodies against mouse IgG (Cat.#. A‐10680, Invitrogen Molecular Probes, Eugene, OR, USA) for 1 h at room temperature. Slides stained with 8‐OHdG were digitized using an Olympus BX53 fluorescent microscope (Olympus America, Center Valley, PA). Lipid peroxidation was measured in homogenized liver tissues based on the reaction between malondialdehyde (MDA) and 2‐thiobarbituric acid (TBA). MDA is an end product of lipid peroxidation, a well‐established biomarker of oxidative stress (Ayala et al., 2014), specifically arising from lipid peroxidation (Yan et al., 2021). The MDA content in liver tissue was determined using a lipid peroxidation (MDA) assay kit (Cat.#. ab118970, Abcam, Cambridge, MA, USA) according to the manufacturer's instructions. Briefly, MDA in liver samples reacted with TBA to generate the MDA‐TBA adduct. The absorbance was measured at 532 nm using a microplate reader, and the MDA content was calculated from the standard curve.

### 2.8 Statistical analysis

The data was analyzed using GraphPad Prism Software version 10.0 (San Diego, CA). Results are presented as means ± standard deviation (SD) and analyzed using a two‐tailed parametric *t‐*test comparing the Control and Obese groups. Sample size (*n*) is indicated for each reported value. Means were statistically significant at *p* < 0.05.

## 3 RESULTS

### 3.1 Western diet leads to central obesity in male mice

**FIGURE 1:**
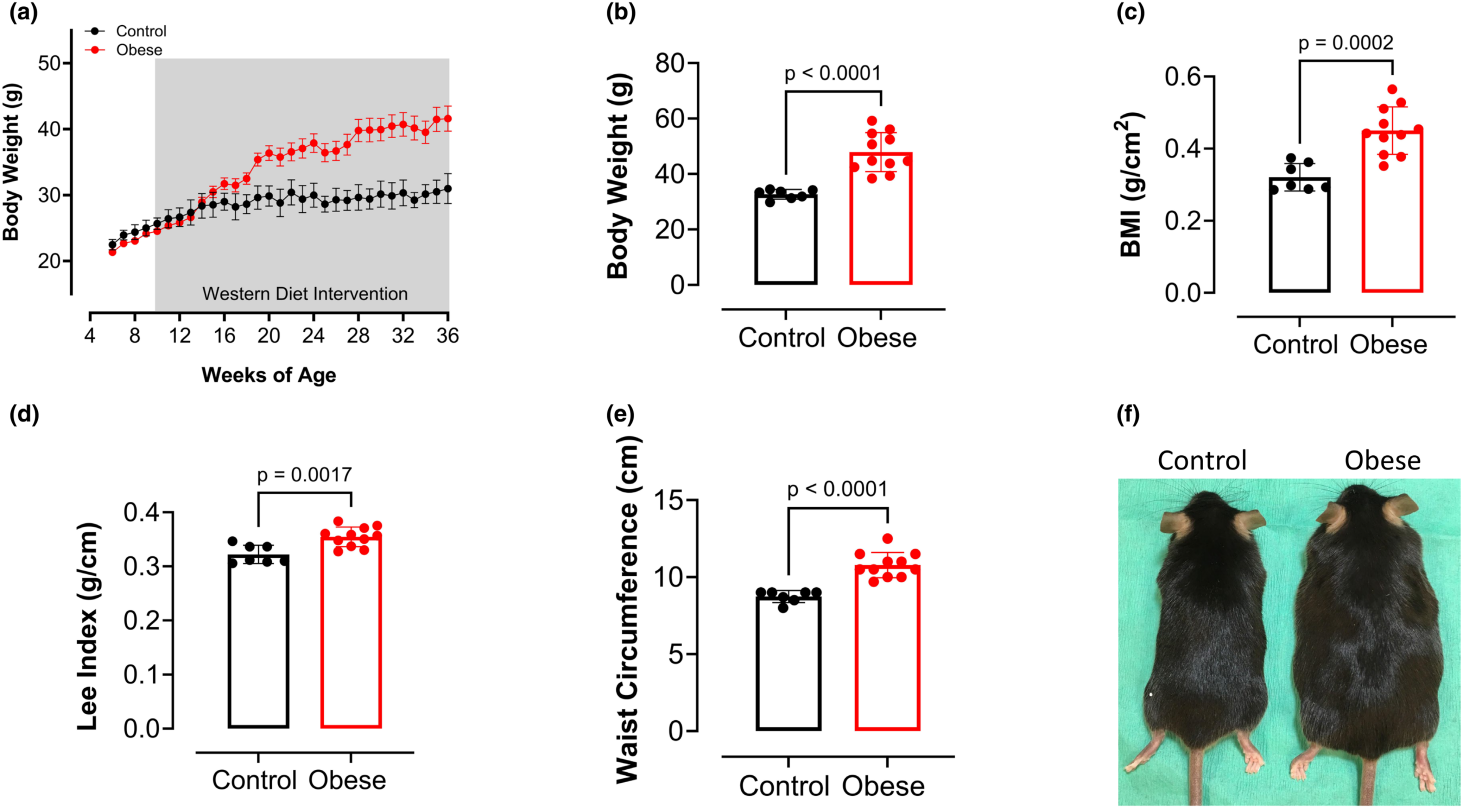
Western diet‐induced obesity in male mice. (a) Body weight (g) of Obese versus Control groups throughout the experimental protocol. (b) Body weight (g), (c) BMI (kg/m^2^), (d) Lee Index (g/cm), and (e) Waist circumference (cm) of obese versus control groups at terminal experiments. (f) Representative photograph of male mice fed standard chow (control group) or Western Diet (obese group). Values are means ± SD. Unpaired *t*‐test (*n* = 7–11 mice/group).

Consumption of a Western diet has been shown to cause weight gain in mice (Bjursell et al., 2008). Body weight was higher in the obese group than in the control group beginning at week 4 on the Western diet (Figure 1a). In the terminal experiments, body weight (Figure 1b), BMI (Figure 1c), and Lee Index (Figure 1d) were significantly higher in the obese group after adherence to 36 weeks of the Western diet. These results were accompanied by a higher waist circumference in the obese group compared to the control group (Figure 1e), characterizing central obesity (Figure 1f).

### 3.2 Obese male mice exhibit intolerance to glucose and insulin resistance

**FIGURE 2:**
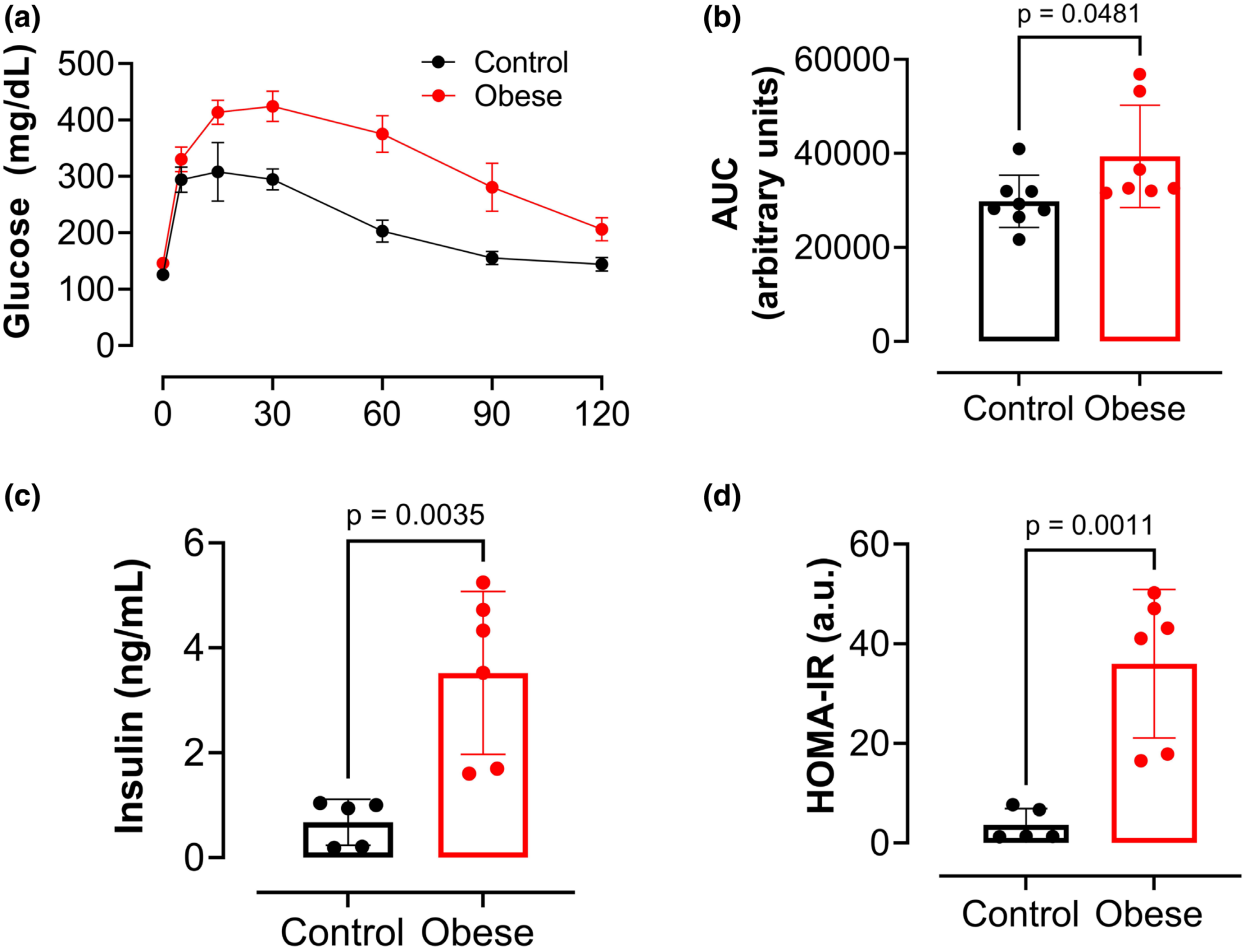
Western diet causes glucose intolerance and insulin resistance in male mice. Western diet‐induced glucose intolerance at 8 weeks on diet. (a) Intra‐peritoneal glucose tolerance test (IPGTT) in Obese versus Control groups. The IPGTT was performed after 8 weeks on diet for 120 min after glucose administration (2 g/kg of body weight). (b) Total blood glucose accumulation is reported as the area under the curve (AUC) of the IPGTT in arbitrary units. (c) Serum insulin levels and (d) HOMA‐IR of obese versus control groups at terminal experiments. Values are means ± SD unpaired *t*‐test (*n* = 5–8 mice/group).

It was evaluated whether mice from the obese group also developed intolerance to glucose and insulin resistance. The obese group exhibited intolerance to glucose after 8 weeks on the Western diet (Figure 2a,b). At the terminal experimental protocol, the obese group showed higher levels of serum insulin (Figure 2c) and HOMA‐IR (Figure 2d) compared to the control group, confirming the presence of insulin resistance in the obese group.

### 3.3 Western diet increases liver weight and liver fat content

**FIGURE 3:**
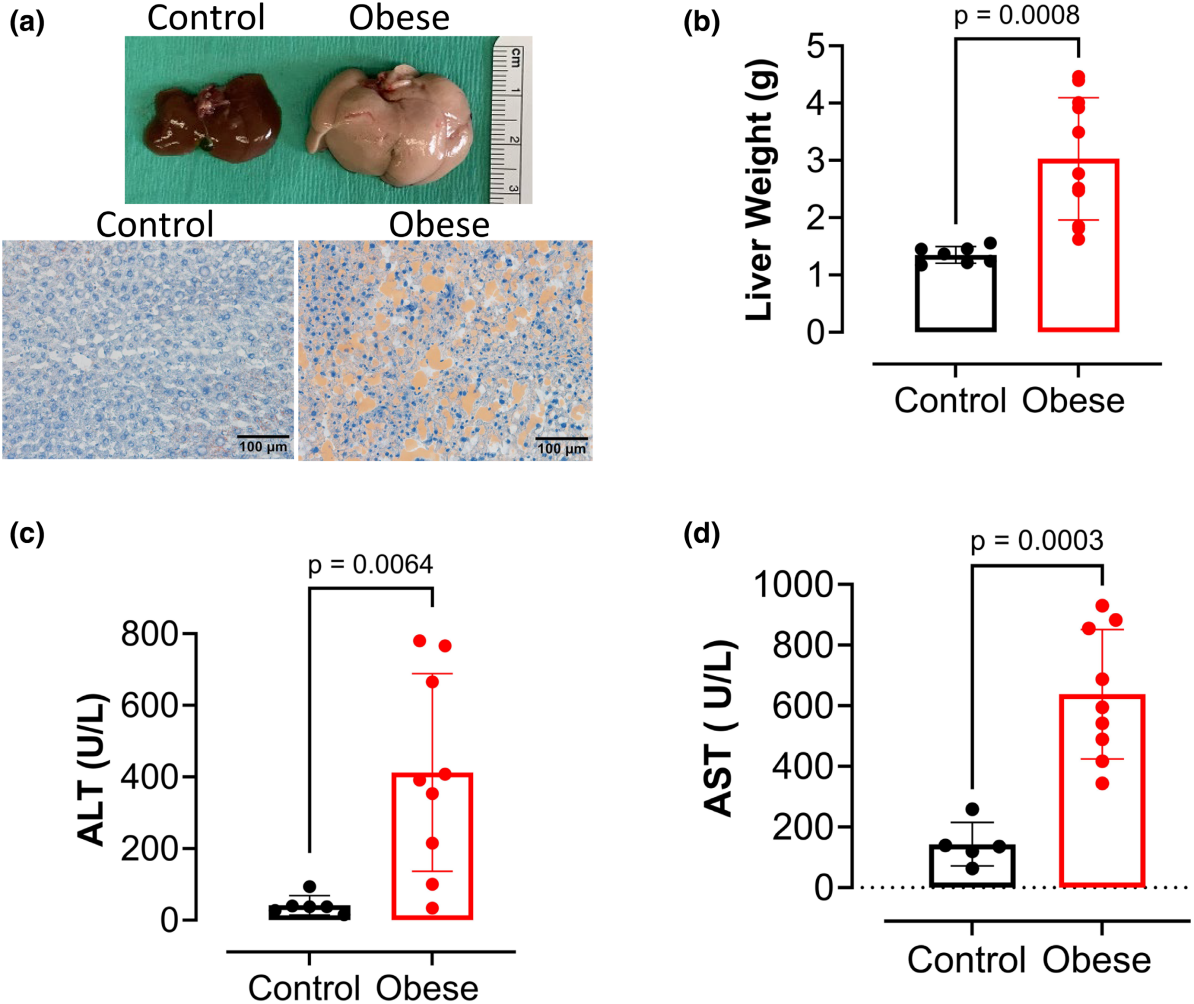
Western diet induces metabolic dysfunction associated with steatotic liver disease in male mice. (a) Representative images of livers and Oil‐Red‐O staining of livers from male mice fed standard chow (control) and Western Diet (obese). (b) Liver weight (g) of Obese versus Control groups. (c) ALT (U/L) and (d) AST (U/L) of Obese versus Control groups. Values are means ± SD unpaired *t*‐test (*n* = 5–9 mice/group).

Mice fed the Western diet exhibited a significant increase in liver weight (Figure 3b). However, the ratio of liver weight to body weight remained unchanged between the two groups (data not shown). The livers from the obese group appeared paler (Figure 3a, top panel). Oil‐red‐stained livers showed that the Obese group exhibited extended steatosis as showed by the presence of more fat droplets (Figure 3a, bottom panel). Additionally, the obese group displayed elevated levels of the liver enzymes ALT and AST (Figure 3c,d).

### 3.4 Western diet promotes metabolic dysfunction associated Steatohepatitis

**FIGURE 4:**
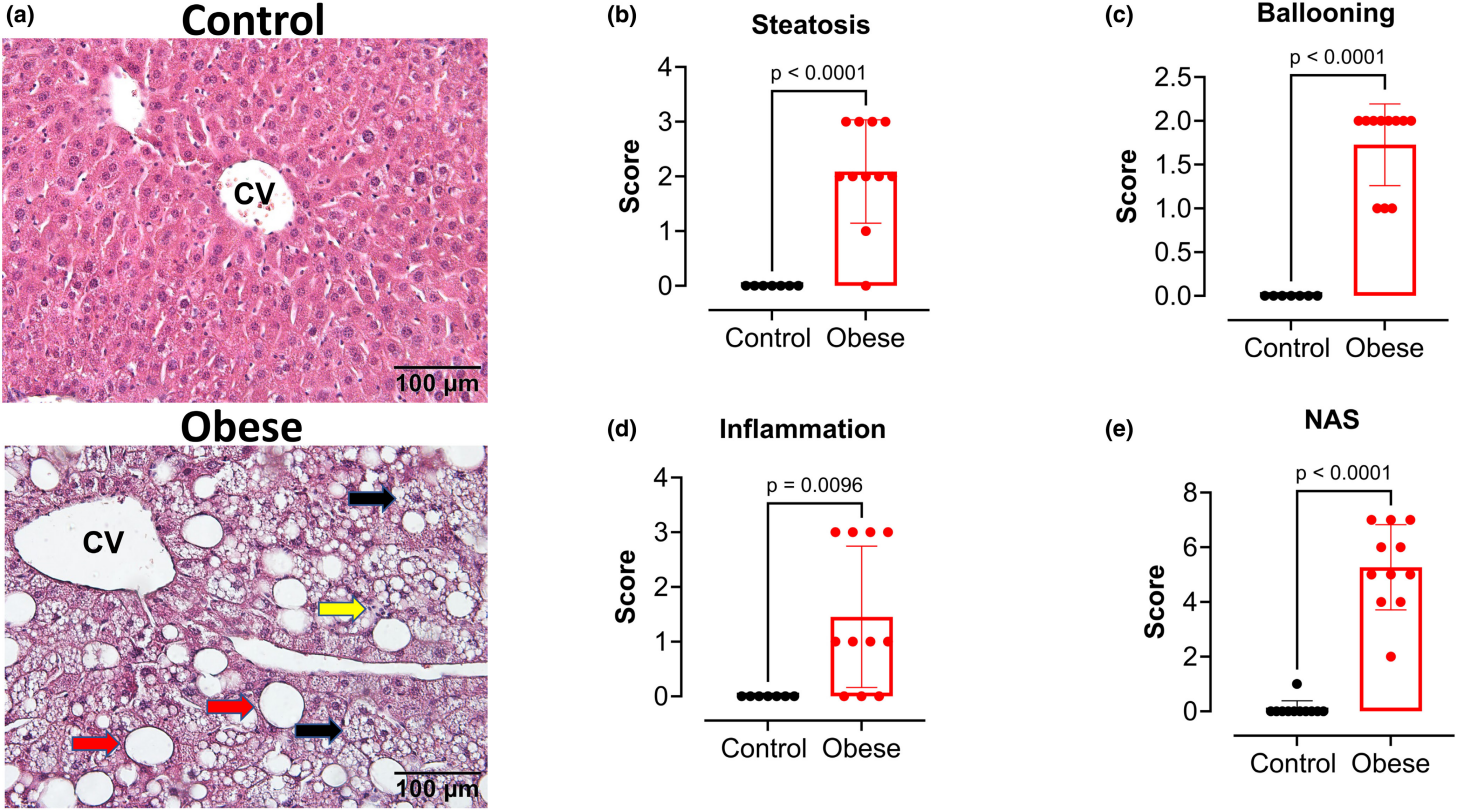
Western diet induces metabolic dysfunction associated with steatohepatitis (MASH) in male mice. NAFLD Activity Score (NAS) is represented as the summation of hepatocyte ballooning (black arrows), Lobular Inflammation (yellow arrows), and Steatosis (red arrows) based on Savari et al. (2019). (a) Representative images of H&E staining of livers of obese and control groups. CV, Central vein. (b) Steatosis score (0–3). (c) Hepatocellular ballooning degeneration score (0–2), (d) Lobular inflammation score (0–3), and (e) NAS in Obese versus Control groups. NAS is the sum of the other scores with a score of 0–2 indicating not MASH, a score of 3–4 indicating borderline MASH, and a score of 5–8 indicating MASH. Values are means ± SD unpaired *t*‐test (*n* = 7–11 mice/group).

Studies have suggested that Western diets can increase the risk of developing MASLD (Zhang, Powell, et al., 2021). To test this theory, we utilized the NAFLD Activity Score (NAS) established by Kleiner et al. H&E‐stained livers from obese and control groups were analyzed to confirm the presence or absence of steatosis, ballooned hepatocytes, and inflammation (Figure 4a) (Kleiner et al., 2005). Livers from the obese group showed a significantly higher percentage of lipid droplets (Figure 4a, bottom panel, red arrows) as compared to controls and were given an average steatosis score of 2–3 (Figure 4b). Ballooned hepatocytes were classified as enlarged hepatocytes with clear cytoplasm and a small amount of stained cytoplasmic filaments. Ballooned hepatocytes were absent in control livers and were assigned an average hepatocyte balloon score of 0. In contrast, livers from the obese group possessed many ballooned hepatocytes (Figure 4a, bottom panel, black arrows) and were given an average score of 2 (Figure 4c). Normal lobular structure, including the absence of lobular inflammation and inflammatory cell infiltration, was observed in the controls, while the obese group showed increased lobular inflammation (Figure 4a, bottom panel, yellow arrow) and was given an average lobular inflammation score of 2 (Figure 4d). This score is representative of moderate to severe inflammatory cell accumulation. The summation of all three scores (hepatocyte ballooning, lobular inflammation, and steatosis) equates to the NAS grade. Obese group scores put them in the MASH category of MASLD, while the control group did not have MASLD (Figure 4e).

### 3.5 Western diet is associated with iron accumulation in the liver

**FIGURE 5:**
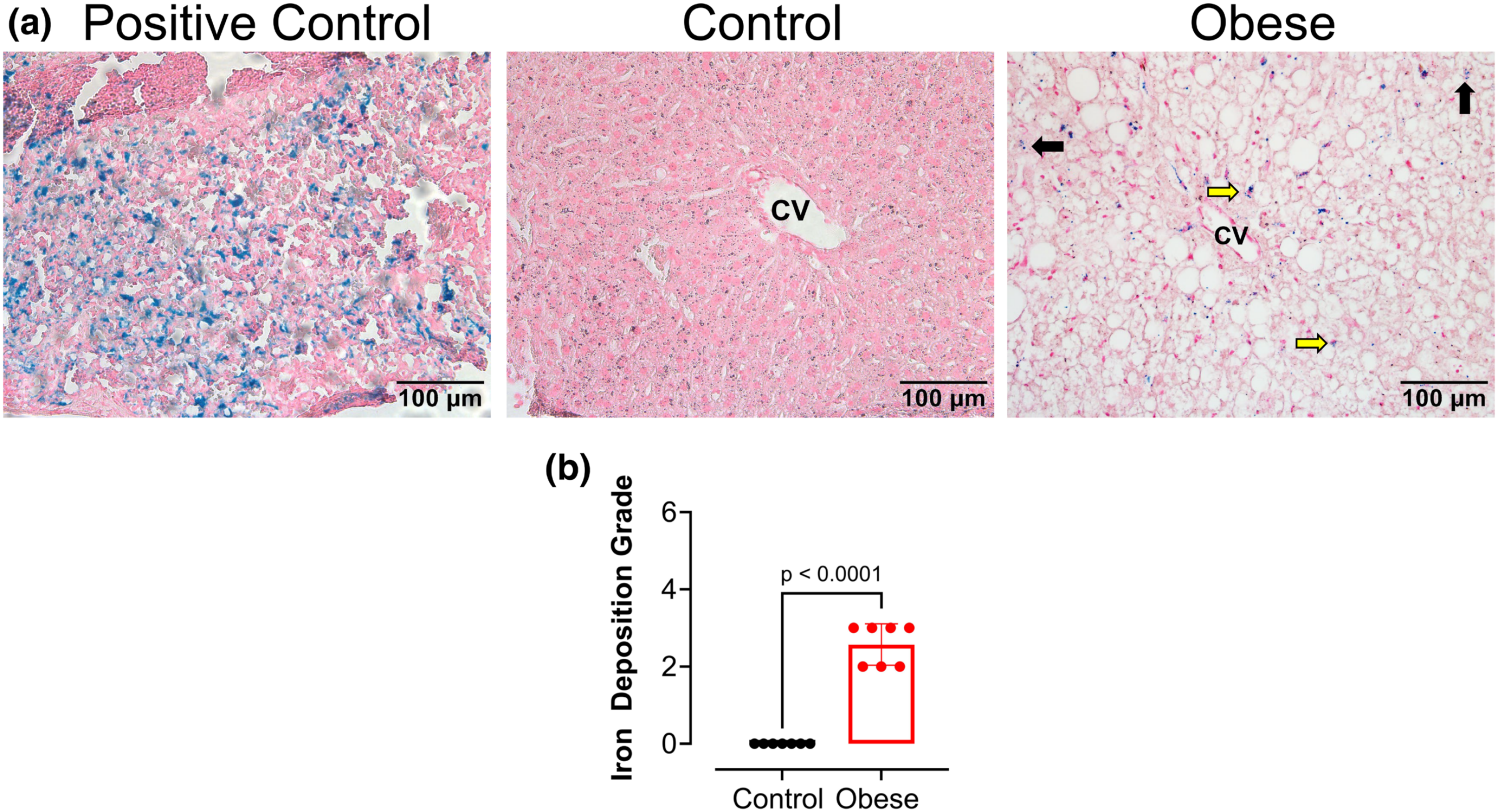
Western diet‐induced MASH exhibits hepatic iron accumulation in male mice. (a) Representative images of iron accumulation on Prussian blue‐stained livers of Obese and Control groups. CV, Central vein. The spleen was used as a positive control. Iron accumulation in Kupffer cells (yellow arrows) and in hepatocytes (black arrows). (b) Grade of hepatic iron staining in the Obese and Control groups. Values are means ± SD unpaired *t*‐test (*n* = 7 mice/group).

Iron accumulation has long been associated with the progression of liver diseases (Kouroumalis et al., 2023). Livers were stained with Prussian blue to visualize iron accumulation. To ensure the staining was successful, an H&E‐stained spleen was utilized as a positive control of the experiment since it naturally has high levels of iron accumulation. Strikingly, the obese group exhibited increased iron accumulation in Kupffer cells and in hepatocytes compared to livers from the control group (Figure 5).

### 3.6 Western diet is associated with increased Ferroptosis markers in the liver

**FIGURE 6:**
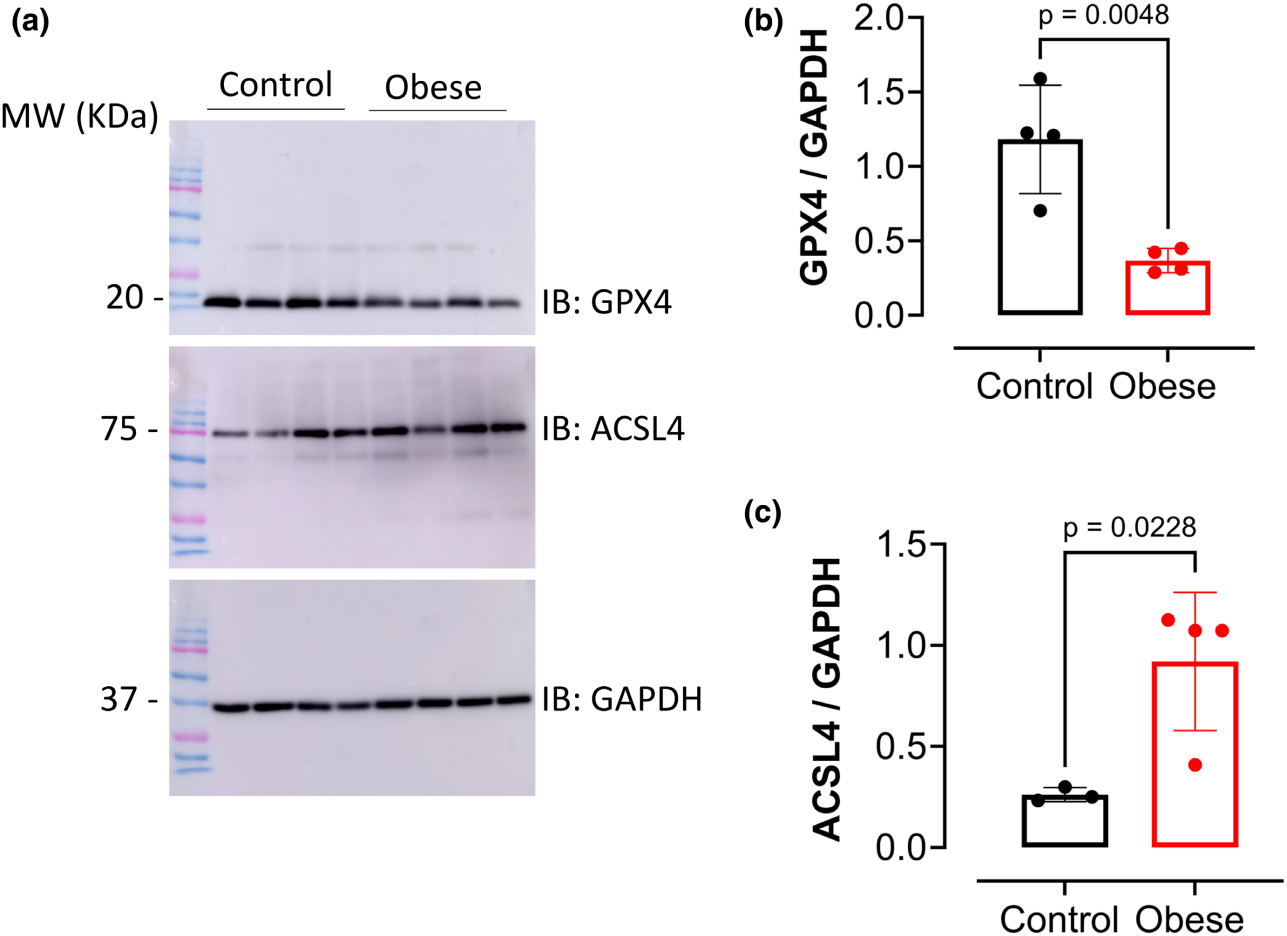
Western diet‐induced MASH exhibits increased ferroptosis markers in the livers of male mice. After the dietary protocol, livers from the Obese and Control groups were analyzed for ferroptosis markers. (a) Representative Western blots for GPX4 and ACSL4 in livers. GAPDH was used as a loading control. (b) Expression of GPX4 and (c) expression of ACSL4 in the liver from Obese versus Control groups. Values are means ± SD unpaired *t*‐test (*n* = 4 mice/group).

Following the identification of increased iron stores in the livers of the obese group, we investigated the expression of markers for ferroptosis (Figure 6a). Of note, GPX4 expression was significantly decreased (Figure 6b), while the expression of ACSL4 was increased in the livers of the obese group (Figure 6c). Together, these results confirm ferroptosis is active in the livers of the obese group.

### 3.7 Western diet causes oxidative hepatic damage

**FIGURE 7:**
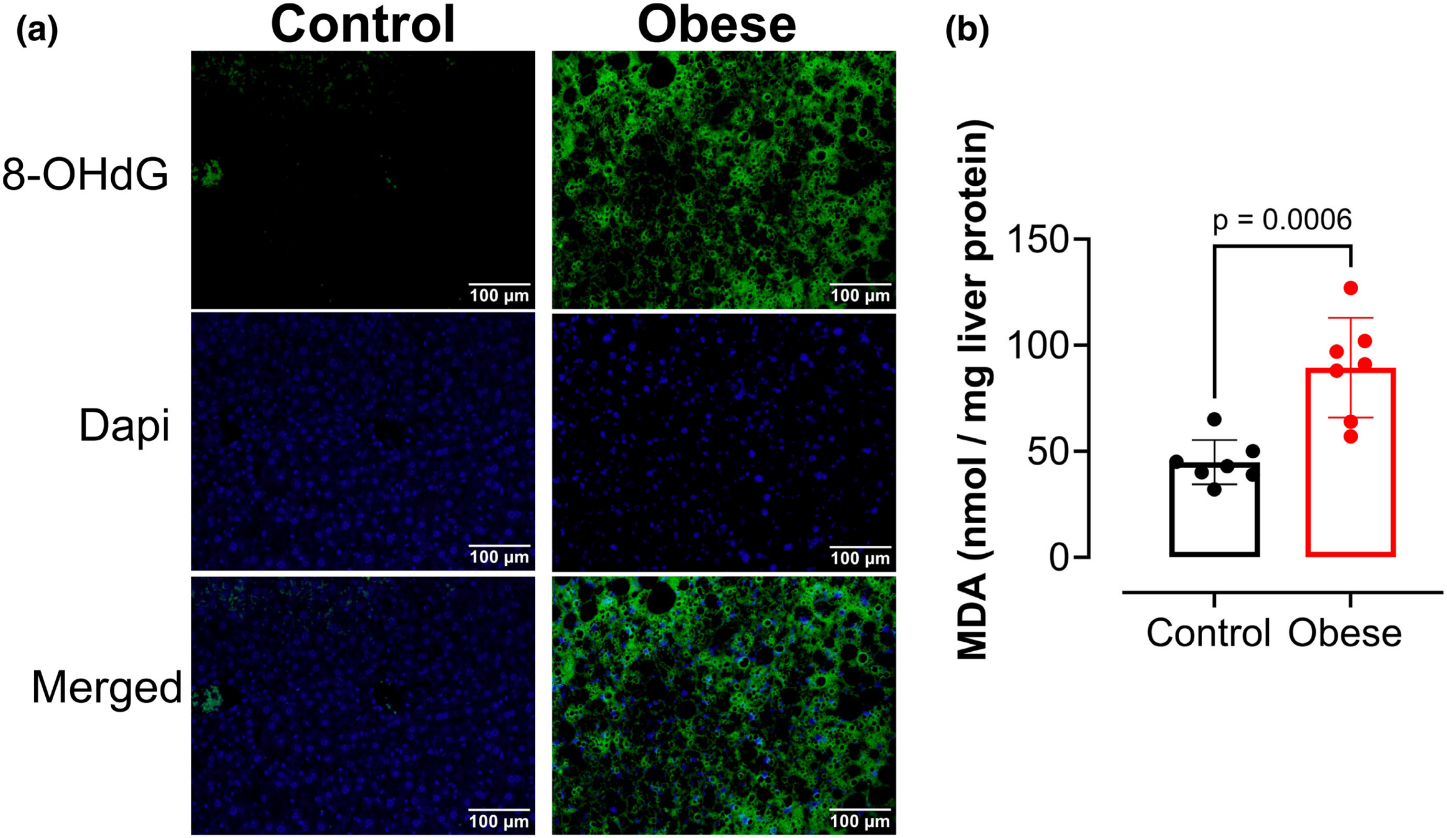
Western diet‐induced MASH exhibits hepatic oxidative damage and increased lipid peroxidation. Livers from the Obese and Control groups were used to measure levels of oxidative damage and lipid peroxidation by using 8‐OHdG staining and MDA levels, respectively. (a) Representative images of 8‐OHdG staining (green) of livers of the Obese and Control groups. Dapi staining nuclei (blue). (b) Levels of MDA in liver lysates from Obese and Control groups. Values are means ± SD unpaired *t*‐test (*n* = 7 mice/group).

Hepatic oxidative damage was assessed by measuring oxidative RNA and DNA damage and lipid peroxidation. Livers from the obese group exhibited increased expression of 8‐OHdG, a marker of oxidative damage (Figure 7a), and increased MDA levels (Figure 7b). Together, these data support that long‐term Western diet consumption leads to hepatic oxidative damage, eliciting ferroptosis.

## 4 DISCUSSION

The key findings of this study include (1) long‐term Western diet‐induced MASLD progressed to MASH in male mice, and (2) progression of MASLD/MASH is associated with hepatic ferroptosis (Abstract Figure). Prolonged Western diet consumption often leads to obesity and MASLD. However, the mechanisms linking Western diet‐induced obesity to MASLD progression remain unclear. This study aimed to identify whether ferroptosis is one of the mechanisms associated with the long‐term Western diet‐induced progression of MASLD. Our results indicate that prolonged Western diet exposure alters the metabolic profile and liver regulatory markers of male mice, thereby predisposing them to MASLD progression, potentially through ferroptosis. These findings are clinically relevant given that MASLD/MASH remains a major health problem in modern society, and there are currently no approved pharmacological therapies or effective treatment options.

Our study confirms that long‐term consumption of a Western diet leads to weight gain and increased BMI in male mice, characterizing obesity. The Western diet‐fed mice weighed significantly more than the standard chow‐fed mice beginning at 6 weeks, and this trend continued until the terminal experiments. The weight gain caused by the Western diet was accompanied by glucose intolerance after 8 weeks, which persisted throughout the entire dietary protocol. Furthermore, the obese mice developed insulin resistance, as evidenced by elevated HOMA‐IR. These results further confirm that long‐term Western diet‐induced obesity impairs glucose metabolism and leads to insulin resistance, processes directly involved in the pathophysiology of MASLD (Sakurai et al., 2021).

It has been well demonstrated that the liver is a key organ negatively affected by diet‐induced obesity (Dai et al., 2018; Lee et al., 2020). Our results show that obese mice exhibited a significant increase in liver mass with pale coloration, indicating an elevated liver fat content that was confirmed by Oil Red‐O staining. Additionally, we observed high levels of ALT and AST, which confirm impaired liver function in the obese mice. These liver regulatory markers have long been linked to increased liver disease mortality and other cardiometabolic risk factors, such as hypertension (Laine et al., 2021; Unalp‐Arida & Ruhl, 2016).

Previous studies examining diet‐induced obesity and MASLD have focused on the effects of short‐term consumption of high‐fat diets, which do not accurately represent the modern Western diet that is high in both fats and carbohydrates. Prior research has shown that long‐term high‐fat diet consumption leads to the development and progression of MASLD to MASH. Importantly, a study by Vacca et al. discussed the various dietary models used to induce MASLD and concluded that a Western diet best mirrors the progression of MASLD and MASH in rodents, as in humans (Vacca et al., 2024), which is in accordance with our results. Additionally, a study by van der Heijden et al. reported the progression of MASLD to MASH in long‐term (40 weeks) high‐fat diet‐fed male mice (Van Der Heijden et al., 2015). The novelty of our results is that the progression of MASLD to MASH developed earlier, at 36 weeks, when the mice were fed a Western diet, indicating that a diet rich in fats and carbohydrates can accelerate the progression of MASLD, and it is associated with hepatic ferroptosis.

The discovery of ferroptosis, a unique form of a nonapoptotic programmed cell death characterized by iron‐dependent lipid peroxidation, has emerged as an essential mechanism involved in a diverse range of pathological conditions (Badgley et al., 2020). This iron‐dependent mechanism causes lipid peroxidation, oxidative stress, and damage characterized by the inactivation or depletion of GPX4, resulting in the accumulation of lipid peroxides and oxidative stress, which triggers ferroptosis (Feng et al., 2022; Jiang et al., 2021; Wang et al., 2020). Previous studies have reported that high levels of iron serve as a driving factor in the induction of ferroptosis, which could further damage liver mitochondria and is associated with high levels of ALT (Wang et al., 2017; Yuan et al., 2023). Clinically, studies have shown that dietary restriction of iron can decrease ALT levels and improve the grade of hepatic iron accumulation in patients with MASLD (Yamamoto et al., 2007). We questioned whether the hepatic iron deposition observed in our obese group was a result of the iron content in the Western diet. Surprisingly, the Western diet used in our study contained much less iron compared to the control diet, which had high iron levels. This confirms that increased hepatic iron deposition in our model of Western diet‐induced obesity and MASLD cannot be attributed to elevated iron consumption. Instead, it suggests that the increased hepatic iron accumulation is likely a consequence of the altered iron metabolism and regulation in the context of obesity and MASLD.

Hepatic iron deposition was accompanied by decreased expression of GPX4 and increased expression of ASCL4 in the livers of obese mice. ASCL4 is an important promoter for ferroptosis, and its increased expression has been linked with lipid peroxidation (Ji et al., 2022; Yuan et al., 2016). To further confirm that decreased GPX4 and increased ASCL4 expression in hepatic tissue from obese mice are linked to active ferroptosis, lipid peroxidation and oxidative damage markers were analyzed. Our results demonstrated that the livers of the Obese group exhibited increased levels of 8‐OHdG, a marker of oxidative damage, and high levels of MDA, a marker of lipid peroxidation, elucidating hepatic damage, which is in accordance with previous studies (Chen et al., 2020; Delli Bovi et al., 2021a, 2021b; Ramos‐Tovar & Muriel, 2020). While oxidative stress and damage can occur by multiple mechanisms in the setting of obesity, our results showing increased iron deposition, decreased GPX4, and increased ASCL4 in the livers of obese mice support ferroptosis as an active mechanism in our model of western diet‐induced obesity and MASLD/MASH in male mice.

It is important to acknowledge the limitations of this investigation. The study subjects used to characterize MASLD/MASH in a model of long‐term Western diet‐induced obesity were male mice. Studies using female C57BL/6J mice are further required to address whether sex‐related differences exist with the prolonged Western diet‐induced MASLD/MASH. Moreover, the utilization of ferroptosis inhibitors, including ASCL4 inhibitors, would further confirm ferroptosis as the main mechanism mediating the progression of MASLD in MASH.

In conclusion, we observed that ferroptosis is present in the livers of obese mice with MASDL/MASH. Ferroptosis can be an important marker and therapeutic target in the investigation of MASLD treatment. Iron chelation therapy can potentially prevent ferroptosis, and future studies can investigate the role of ferroptosis in MASLD treatment.

## FUNDING INFORMATION

The National Institutes of Health (NIH) National Heart, Lung, and Blood Institute (NHLBI) Grant/Award R15 HL 165328 to MACS. N. Maddie holds a 2022 Scholarship in Cardiovascular Diseases from the American Heart Association.

## CONFLICT OF INTEREST STATEMENT

The authors declare no conflict of interest.

## ETHICS STATEMENT

All preclinical experimental protocols followed the Animal Research Reporting of in Vivo Experiments (ARRIVE) guidelines and were conducted in accordance with the Institutional Animal Care and Use Committee at the New York Institute of Technology College of Osteopathic Medicine and with respect to the National Institute of Health Guidelines for the Care and Use of Laboratory Animals.

## Data Availability

All data generated and analyzed during the current study are available from the corresponding author upon reasonable request.
